# Combination of Intravesical Bacille Calmette-Guérin and Chemotherapy vs. Bacille Calmette-Guérin Alone in Non-muscle Invasive Bladder Cancer: A Meta-Analysis

**DOI:** 10.3389/fonc.2019.00121

**Published:** 2019-03-01

**Authors:** Di Huang, Ying-Hui Jin, Hong Weng, Qiao Huang, Xian-Tao Zeng, Xing-Huan Wang

**Affiliations:** ^1^Center for Evidence-Based and Translational Medicine, Zhongnan Hospital of Wuhan University, Wuhan, China; ^2^Department of Urology, Zhongnan Hospital of Wuhan University, Wuhan, China

**Keywords:** non-muscle invasive bladder cancer, bacille Calmette-Guérin, chemotherapy, prognosis, meta-analysis

## Abstract

**Background:** About 75% of newly diagnosed bladder cancer cases suffer from non-muscle invasive bladder cancer (NMIBC), which used to recur and progress despite transurethral resection of bladder tumor (TURBT). This meta-analysis was conducted to examine if combined application of intravesical bacille Calmette-Guérin (BCG) with chemotherapy is associated with better prognosis.

**Methods:** Systematic searches of randomized controlled trials (RCTs) concerning NMIBC were performed in PubMed, EMbase, CENTRAL, CNKI, WanFang, VIP, CBM databases, and some specialized websites. Two researchers independently implemented study selection, quality assessment and data extraction. Hazard ratios (HRs) and their 95% confidence intervals (CIs) for treatment effects on recurrence-free survival (RFS), progression-free survival (PFS), overall survival (OS) and disease-specific survival (DSS) were directly extracted, if available, or estimated using relevant data from included studies. Side effects, such as fever, gastrointestinal reaction, cystitis, irritative bladder symptoms and hematuria, were also extracted as outcome measurements, and associated relative risks (RRs) were calculated to assess treatment safety. RevMan 5.3 software was used to perform statistical analyses.

**Results:** Thirteen RCTs containing 1,754 patients with NMIBC were included in this meta-analysis. Compared with BCG alone, the combination therapy significantly improved RFS (HR = 0.53, 95% CI: 0.43–0.66, *P* < 0.01), OS (HR = 0.66, 95%CI: 0.50–0.86, *P* = 0.002), and DSS (HR = 0.48, 95%CI: 0.29–0.80, *P* = 0.005). While PFS showed no obvious difference between combination therapy and BCG alone (HR = 0.65, 95%CI: 0.25–1.68, *P* = 0.38). The rate of fever (RR = 0.50, 95%CI: 0.27–0.91, *P* = 0.02), irritative bladder symptoms (RR = 0.69, 95%CI: 0.52–0.90, *P* = 0.007) and hematuria (RR = 0.50, 95%CI: 0.28–0.89, *P* = 0.02) were significantly decreased in patients treated with combination therapy compared to those with BCG alone. There were no statistically significant differences between combination therapy and BCG alone in toxicity (RR = 0.69, 95%CI: 0.34–1.40, *P* = 0.30), gastrointestinal reaction (RR = 2.54, 95%CI: 0.61–10.60, *P* = 0.20) or cystitis (RR = 0.67, 95%CI: 0.29–1.54, *P* = 0.34).

**Conclusions:** Combined application of intravesical BCG and chemotherapy appears to be an effective treatment for patients with intermediate- to high-risk NMIBC, but not for those with tumor *in situ* alone or recurrent bladder cancer.

## Introduction

In terms of its popularity, bladder cancer ranks the fourth and the 11 places among men and women, respectively, with more than 430,000 new cases annually worldwide (1). Approximately 75% of newly diagnosed bladder cancer cases belong to non-muscle invasive bladder cancer (NMIBC), of which Ta accounts for 60%, T1 for 30% and Tis for 10% (2–6). Although prognosis of patients with NMIBC is greatly improved, the treatment for the malignancy still faces serious problems: recurrence and progression (7). Of NMIBC patients, 60 to 70% will recur while 20 to 30% will progress into higher stages (6). Thus, intensive treatment and surveillance create a considerable economic burden on both countries and individuals.

The mainstay for treating NMIBC is transurethral resection of bladder tumor (TURBT) followed by adjuvant intravesical therapies, primarily including bacille Calmette-Guérin (BCG) immunotherapy (6). Although BCG has been proved to be effective in preventing tumor recurrence and progression, not all NMIBC patients could receive it due to considerable side effects, which severely compromise its therapeutic effects (2, 8, 9). Since a large proportion of NMIBC patients poorly tolerate BCG, there is a pressing need for alternative treatments. The risk of recurrence could be decreased via intravesical chemotherapy, but with weaker effect than BCG (10). A treatment combing intravesical BCG with chemotherapy has been developed to enhance BCG effects and to alleviate side effects. With lowered BCG dose, patients are more likely to accept the combination of intravesical BCG and chemotherapy, thus reducing side effects. Meanwhile, immunological response of BCG will be evoked via the anti-tumor mechanism of chemotherapy, thereby possibly potentiating BCG effect, and therefore, an optimized treatment regimen will finally be achieved (7). Unfortunately, such combinations are not always related to significantly decreased recurrence or progression in trails (7). A meta-analysis conducted in 2013 showed that the combination possessed no superiority over BCG alone among NMIBC patients (11). In addition, the efficacy of combing intravesical BCG with chemotherapy in treating NMIBC remains poorly understood, and clinical experience of this approach is still limited by far. With more trials on combination therapy emerging, interest in its efficacy has been strengthened. The aim of this meta-analysis is to discuss whether combination therapy is associated with better efficacy and less side effects than BCG monotherapy.

## Methods

### Eligibility Criteria

Studies were eligible for inclusion if they met the following criteria: (1) participants: NMIBC patients receiving TURBT; (2) intervention: intravesical BCG plus chemotherapy; (3) control: BCG alone; (4) containing at least one of the following outcomes: recurrence-free survival (RFS) (time interval from randomization to bladder tumor recurrence), progression-free survival (PFS) (time interval from randomization to entering into higher bladder tumor stages), overall survival (OS) (time interval from randomization to death due to any causes), disease-specific survival (DSS) (time interval from randomization to death caused only by bladder cancer), and side effects (fever, gastrointestinal reaction, cystitis, irritative bladder symptoms, hematuria, etc.). Only randomized controlled trials (RCTs) comparing the efficacy or safety of intravesical BCG plus chemotherapy with BCG alone were included. Studies were excluded for the below reasons: (1) patients with Tis alone or recurrent bladder cancer; (2) data could not be obtained even after contacting original author; (3) duplicated publications. When multiple studies were delivered by the same team based on similar patients, only the largest or the most comprehensive one was included.

### Study Search and Selection

Trials focusing on NMIBC were identified through searching PubMed, EMbase, CENTRAL, CNKI, WanFang, VIP, and CBM databases. ClinicalTrials.gov (https://www.clinicaltrials.gov/), the World Health Organization International Clinical Trials Registry Platform (WHO ICTRP, http://www.who.int/ictrp/en/), American Urological Association (http://www.auanet.org/), European Association of Urology (http://uroweb.org/), American Society of Clinical Oncology (https://www.asco.org/) and the Gray Literature Report (http://www.greylit.org/) were also searched to identify ongoing and unpublished trials. We also screened reference lists of included studies, relevant articles and meta-analyses in this field to find any other qualified articles. Considering that all the articles concentrated on surgical treatment, chemotherapy, immunotherapy, combination therapy, carcinoma *in situ*, and follow-up of NMIBC would be reviewed by our team members, respectively. Here we designed the search strategy for RCT on NMIBC. The following keywords and medical subject headings (MeSH) terms were used in combination: “bladder” or “transitional cell” or “urothelial” or “urothelium” or “urinary bladder” or “upper tract urothelial,” “cancer” or “carcinoma” or “tumor” or “neoplasm,” “non-muscle invasive” or “superficial” or “early” or “Ta” or “T1” or “Tis” or “CIS,” and “randomized controlled trial” or “RCT” or “clinical trial.” The search was limited to studies on humans without language restriction.

Titles and abstracts were examined by two independent reviewers (DH and Y-HJ) according to aforementioned eligibility criteria. After removing duplicated and irrelevant studies, remaining studies were underwent full-text examination. Disagreements were resolved through discussion with a third reviewer (HW).

### Quality Assessment and Data Extraction

The quality of all included studies was assessed using the “risk of bias” tool recommended by the Cochrane Collaboration. This tool consists seven domains: random sequence generation, allocation concealment, blinding of participants and personnel, blinding of outcome assessment, incomplete outcome data, selective reporting and other bias (12). Two reviewers (DH and Y-HJ) independently evaluated the quality of studies in these domains. Finally, each study was classified into one of three categories: low risk, unclear risk or high risk.

Data extraction was independently undertaken by 2 reviewers (DH and Y-HJ). The following information was extracted: first author's name, year of publication, study period, country of study, sample size, sex and age of participants, stage and risk of bladder tumor, treatment duration, follow-up duration, treatment schedule, and relevant data on outcomes. Disagreements were discussed until a final consensus was finally achieved.

### Statistical Analysis

Hazard ratios (HRs) and associated 95%confidence intervals (CIs) were used to assess the effect of combination therapy on prognosis in patients with NMIBC (13). If HRs and their 95%CIs were not reported in original articles, we could obtain approximate estimates using methods proposed by Tierney et al. (14, 15). Moreover, Relative risks (RRs) with 95%CIs for side effects were calculated to evaluate the safety of combination therapy. Chi-squared tests were used to detect heterogeneity between studies included in this meta-analysis. Considering that the statistical power of heterogeneity test is generally low, a *P*-value of 0.10 was set as the significance threshold for the heterogeneity. When *P* ≤ 0.1, heterogeneity was significant. We used I-squared (*I*^2^) statistic to indicate the proportion of variation between the studies due to heterogeneity. The larger the *I*^2^ value represented, the higher the heterogeneity was. And *I*^2^ > 50% suggested substantial heterogeneity among the studies. Fixed effect model was adopted when no significant heterogeneity was detected (*P* > 0.1 and *I*^2^ < 50%), otherwise, random effect model would be used. Planned subgroup analyses were conducted to assess the difference between two treatment regimens according to the country of study (China vs. foreign countries), chemotherapeutic agent used in combination therapy (mitomycin C (MMC), epirubicin (EPI), pirarubicin (THP)), treatment duration (>1 year vs. ≤1 year), pathological risk (with tumor *in situ* (Tis) vs. without Tis) and risk level (high, intermediate, low). All statistical analyses were performed using RevMan software (version 5.3, The Cochrane Collaboration).

## Results

### Study Selection

**Figure 1:**
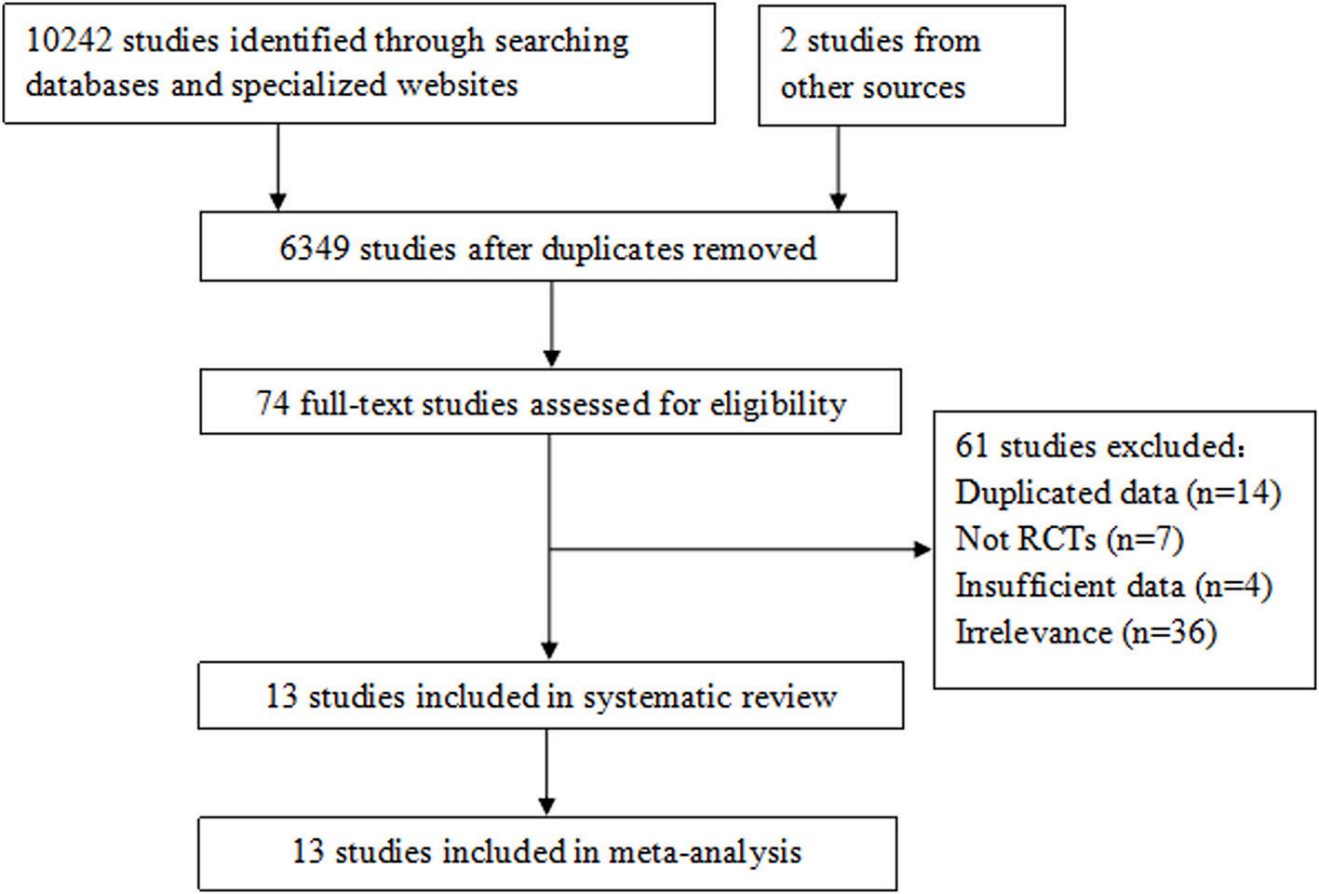
Flow diagram of study selection in the meta-analysis.

Ten thousand two hundred and forty-four studies were identified from the electronic databases, specialized websites and other sources. Three thousand eight hundred and ninety-five duplicated studies were first removed. After screening titles and abstracts, we obtained 74 potentially relevant studies, and their full texts were carefully read for eligibility examination, in which 61 studies were eliminated for duplicated data (14), not RCTs (7), lacking sufficient data to estimate HRs or RRs of outcome measurements (4), and irrelevance (36). Finally, 13 studies with a total of 1,754 participants were included in the meta-analysis (16–28). The process of study selection is shown in Figure 1.

### Study Characteristics

The characteristics of the included studies are summarized in Table 1. These studies were published between 1999 and 2015, with an RCT design. Six studies were performed in China, 2 in Italy, 2 in Turkey, 2 in Egypt, and 1 in Spain. Totally, 1,754 participants were enrolled, with a median size of 124 (ranging from 51 to 407). All of the studies enrolled patients with NMIBC and 4 of them included patients with concurrent Tis. The patients were reported to have high-risk NMIBC in 5 studies and intermediate- to high-risk NMIBC in two studies. Treatment duration was more than 1 year in five studies and equal to or <1 year in the other eight ones. The median duration of follow-up across the studies was 24 months. Treatment schedules varied between these studies.

**Table 1:** Characteristics of the studies included in the meta-analysis.

**First author, year**	**Study period**	**Country**	**Sample size**	**Sex**	**Age**	**Stage**	**Risk level**	**Treatment duration**	**Follow-up**	**Outcomes**	**Treatment schedule**
Pei (16)	2010–2013	China	163	103/60	66/58	Ta, T1, Tis	–	>1 year	2 years	5, 7, 8, 9, 10	BCG+THP: 6 days after TURBT, alternating weekly THP (50 mg) and BCG (120 mg) for 8 weeks + alternating monthly THP and BCG for 2 years; BCG: 6 days after TURBT, weekly BCG (120 mg) for 8 weeks + monthly BCG for 2 years.
Di Stasi et al. (17)	1994–2002	Italy	212	173/39	66	T1	High	≤1 year	121 (70.5–163.5)m	1, 2, 3, 4	BCG+MMC: weekly BCG (81 mg) and MMC (40 mg) for 9 weeks (1 cycle = 2 BCG + 1 MMC) + disease-free 3 months after treatment, monthly BCG and MMC for 9 months (3 cycles of MMC, MMC and BCG); BCG: weekly BCG (81 mg) for 6 weeks + disease-free 3 months after treatment, monthly BCG for 10 months.
Solsona et al. (18)	1993–1994	Spain	407	366/41	67	Ta, T1, Tis	High	≤1 year	7.1 years	1, 2, 3, 4, 5	BCG+MMC: weekly BCG (81 mg) + MMC (30 mg) for 6 weeks followed by 3 more instillations 2 weeks apart, the first instillation between 14 and 28 days after TURBT; BCG: weekly BCG (81 mg) for 6 weeks followed by 3 more instillations 2 weeks apart.
Gong et al. (19)	2008–2010	China	95	79/16	53–75	Ta, T1	–	>1 year	3 year	5	BCG+MMC: 2 weeks after TURBT, alternating weekly MMC (40 mg) and BCG (80 mg) for more than 8 weeks + MMC and BCG every 2 weeks for 3 months + monthly MMC and BCG for 8 months; BCG: 2 weeks after TURBT, weekly BCG (120 mg) for more than 8 weeks + BCG every 2 weeks for 3 months + monthly BCG for 8 months.
He et al. (20)	2005–2009	China	79	62/17	–	–	–	≤1 year	(21.2 ± 9.6)m	5, 6, 7, 9, 10	BCG+MMC: MMC (40 mg) within 6 h of surgery + weekly BCG for 6 weeks + monthly BCG for 12 months (if no recurrence occurs, BCG every 3 months for 9 months); BCG: 7 days after TURBT, weekly BCG for 6 weeks + monthly BCG for 12 months (if no recurrence occurs, BCG every 3 months for 9 months).
Gulpinar et al. (21)	2004–2006	Turkey	51	41/10	–	Ta, T1, Tis	Intermediate to high	≤1 year	41.3(8–64) m 40.9 (6–68) m	1, 10	BCG+MMC: MMC (40 mg) within 6 h of surgery followed by weekly BCG (5 × 10^8^ CFU) for 6 weeks at least 15 days from TURBT; BCG: weekly BCG (5 × 10^8^ CFU) for 6 weeks.
El Kader et al. (22)	2004–2008	Egypt	128	86/42	54	Ta, T1	–	≤1 year	26 (6–45) m	6, 9, 10	BCG+MMC: weekly MMC (40 mg) + BCG for 6 weeks 3 weeks post TURBT; BCG: weekly BCG for 6 weeks 3 weeks post TURBT.
Jiang et al. (23)	2005–2006	China	55	43/12	52	Tis, T1	Intermediate to high	>1 year	24 m	6, 9, 10	BCG+MMC: 1 week after TURBT, alternating weekly MMC (20 mg) and BCG (120 mg) for 3 months + alternating MMC and BCG every 2 weeks for 3 months + alternating MMC and BCG every 6 months for 2 years; BCG: 1 week after TURBT, weekly BCG (120 mg) for 6 weeks + monthly BCG for 2 years.
Song et al. (24)	2004–2009	China	128	97/31	57.1	–	–	≤1 year	52.4 (18–60) m	6, 9	BCG+THP: 1 week after TURBT, alternating weekly THP and BCG for 8 weeks + alternating weekly THP and BCG for 8 months; BCG: 1 week after TURBT, weekly BCG for 8 weeks + weekly BCG for 8 months.
Cai et al. (25)	2005–2007	Italy	161	–	–	Ta, T1	High	>1 year	15.3 (3–30) m 14.8(4–27) m	1, 10	BCG+EPI: EPI (80 mg) within 6 h of surgery + weekly BCG (5 × 10^8^ CFU) for 6 weeks at least 21 days from TURBT + BCG at 3, 6, 12, 18, 24, 30, and 36 months; BCG: weekly BCG (5 × 10^8^ CFU) for 6 weeks at least 21 days from TURBT + BCG at 3, 6, 12, 18, 24, 30, and 36 months.
Liu et al. (26)	2000–2003	China	110	84/26	55	Ta, T1	–	>1 year	35 (12–70) m	8	BCG+MMC: MMC (20 mg) within 6 h of surgery + weekly BCG (150 mg) for 6 weeks 3 weeks after TURBT + weekly BCG at 3, 6, 12, 18, 24, 30, and 36 months; BCG: weekly BCG (150 mg) for 6 weeks 3 weeks after TURBT + weekly BCG at 3, 6, 12, 18, 24, 30, and 36 months.
Bilen et al. (27)	1994–1995	Turkey	41	39/2	–	T1	High	≤1 year	18 (9–24)m	6, 8, 9, 10	BCG+EPI: 10–15 days following TURBT, weekly sequential BCG (81 mg) and EPI (50 mg) for 1 year; BCG: 10–15 days following TURBT, weekly BCG (81 mg) for 6 weeks.
Ali-El-Dein et al. (28)	1993–1997	Egypt	124	96/28	58.2	Ta, T1, Tis	High	≤1 year	30.4 (12–50) m	1, 5, 6, 8, 10	BCG+EPI: alternating weekly BCG (150 mg) + EPI (50 mg) for 6 weeks and monthly for 10 months 1 to 3 weeks after TURBT; BCG: weekly BCG (150 mg) for 6 weeks and monthly for 10 months 1 to 3 weeks after TURBT.

–*, Not report; THP, pirarubicin; MMC, mitomycin C; EPI, epirubicin; 1, Recurrence-free survival (RFS); 2, Progression-free survival (PFS); 3, Overall survival (OS); 4, Disease-specific survival (DSS); 5, Toxicity; 6, Fever; 7, Gastrointestinal reaction; 8, Cystitis; 9, Irritative bladder symptoms; 10, Hematuria*.

### Quality Assessment

**Figure 2:**
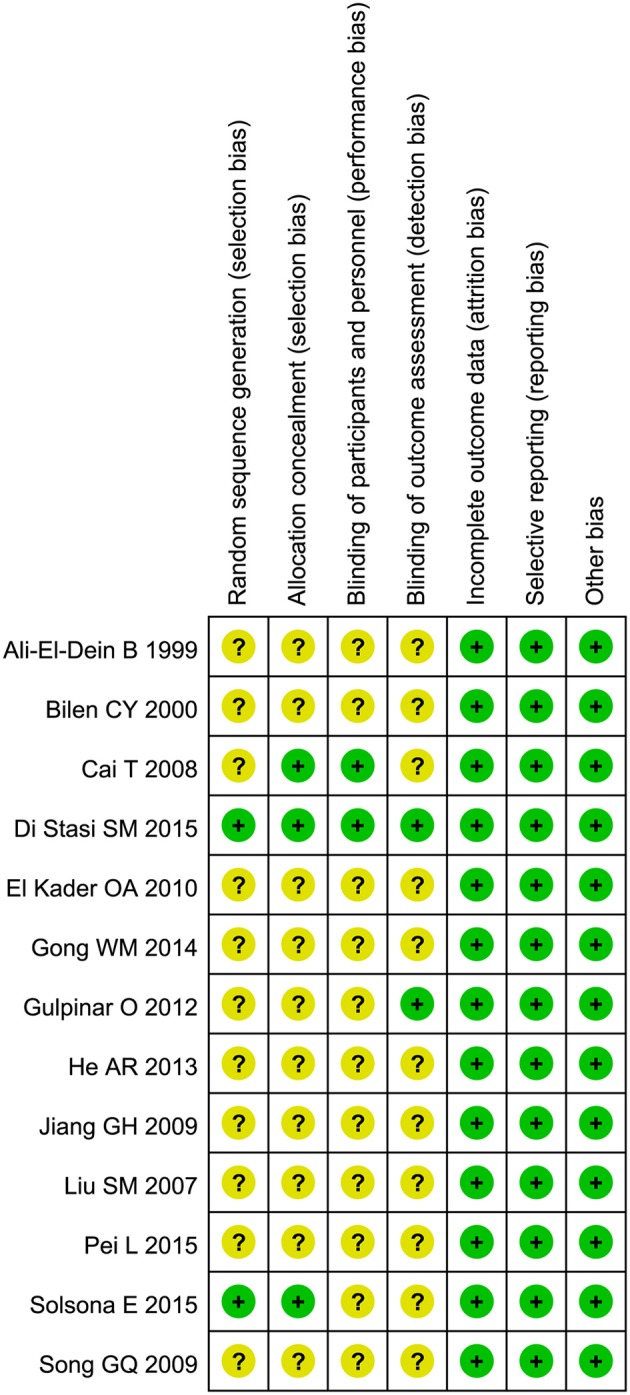
Risk of bias assessment of included studies.

The “risk of bias” tool recommended by the Cochrane Collaboration was adopted to assess the quality of all included studies (Figure 2). Two studies (17, 18) described how random sequence was generated, and three (17, 18, 25) described the method of allocation concealment. Blind method was mentioned in only two studies (17, 25). No incomplete or selective outcome data was reported.

### Recurrence-Free Survival

**Figure 3:**
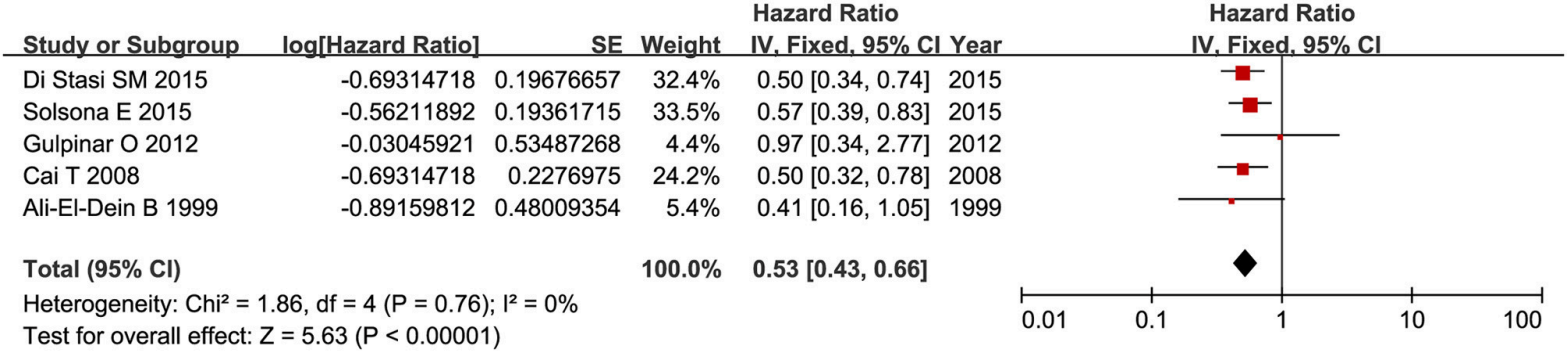
Forest plot of RFS comparing combination therapy with BCG alone.

**Figure 4:**
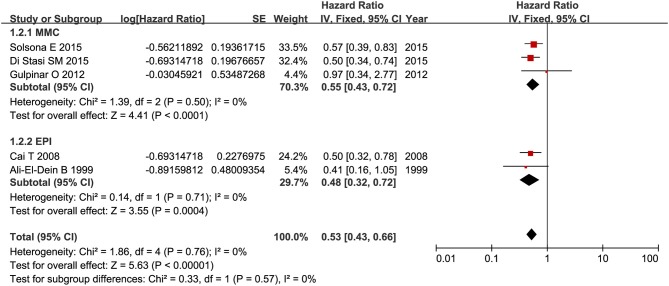
Comparison on RFS between combination therapy and BCG alone after subgroup analysis stratified by chemotherapeutic agent used in combination therapy.

**Figure 5:**
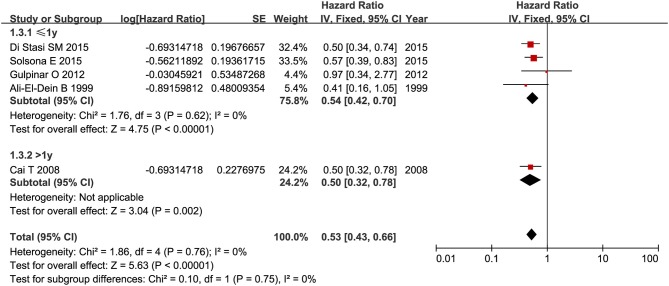
Comparison on RFS between combination therapy and BCG alone after subgroup analysis stratified by treatment duration.

**Figure 6:**
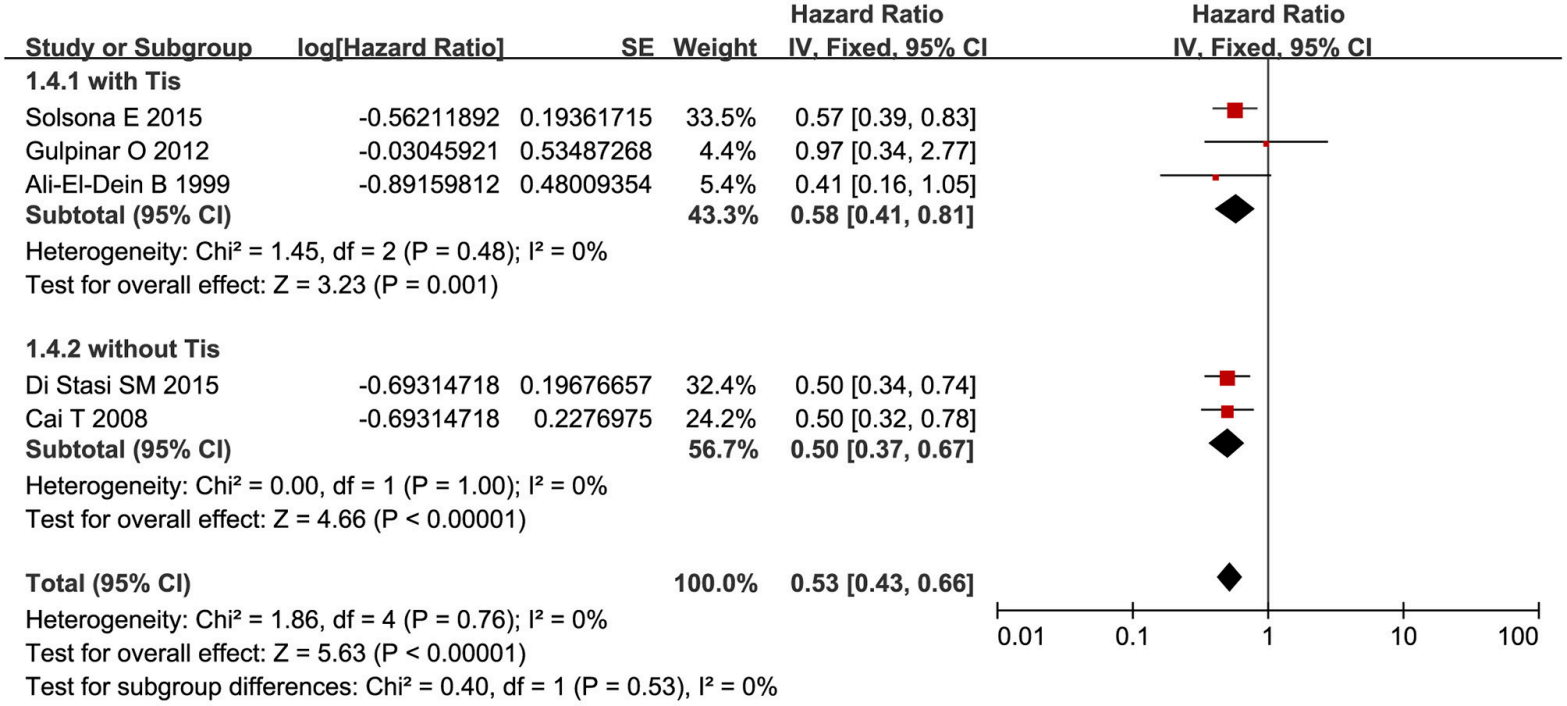
Comparison on RFS between combination therapy and BCG alone after subgroup analysis stratified by pathological risk of patients.

**Figure 7:**
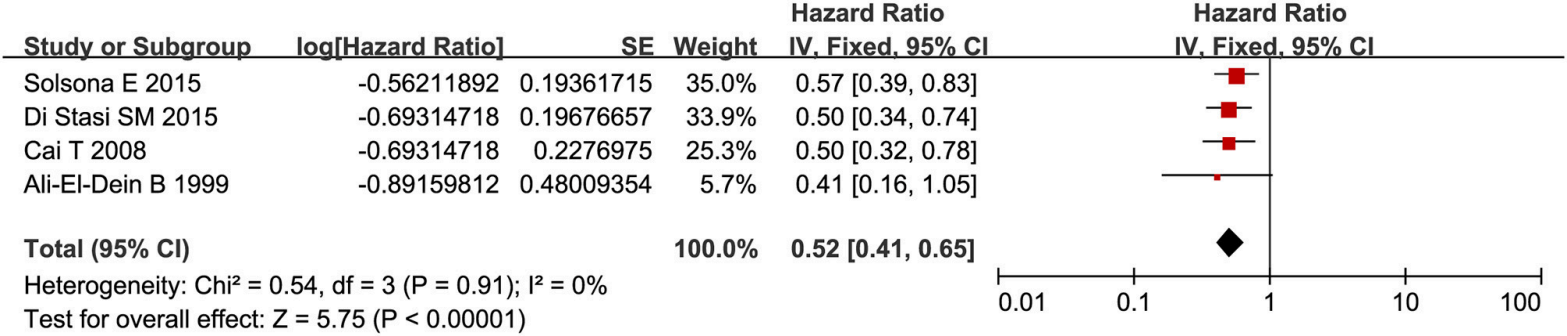
Comparison on RFS between combination therapy and BCG alone in patients with high-risk NMIBC.

RFS was compared between combination therapy and BCG alone in five studies (*n* = 955) (17, 18, 21, 25, 28). And pooled HR exhibited substantial advantage for combination therapy over BCG alone (HR = 0.53, 95%CI: 0.43–0.66, *P* < 0.01) without significant heterogeneity (*P* = 0.76, *I*^2^ = 0%), indicating a 47% reduction in the risk of tumor recurrence (Figure 3). Due to the small number of relevant studies, funnel plot could not detect publication bias. Subgroup analysis according to chemotherapeutic agent used in combination therapy showed better effect for combination therapy with either MMC (HR = 0.55, 95%CI: 0.43–0.72, *P* < 0.01) or EPI (HR = 0.48, 95%CI: 0.32–072, *P* < 0.01) (Figure 4). After stratified by treatment duration (>1 year vs. ≤1 year), combination therapy significantly reduced the risk of recurrence compared with BCG alone regardless of treatment duration (≤1 year: HR = 0.54, 95%CI: 0.42–0.70, *P* < 0.01; >1 year: HR = 0.50, 95%CI: 0.32–0.78, *P* = 0.002) (Figure 5). Subgroup analysis showed a statistically significant benefit from combination therapy compared with BCG alone in patients with concurrent Tis (HR = 0.58, 95%CI: 0.41–0.81, *P* = 0.001) or not (HR = 0.50, 95%CI: 0.37–0.67, *P* < 0.01) (Figure 6). Patients with high-risk NMIBC who underwent combination therapy achieved a significantly better RFS than those who underwent BCG alone (HR = 0.52, 95%CI: 0.41–0.65, *P* < 0.01) (Figure 7).

### Progression-Free Survival

**Figure 8:**
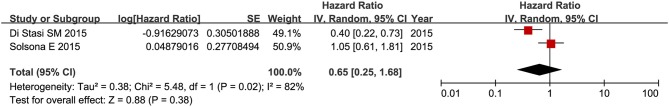
Forest plot of PFS comparing combination therapy with BCG alone.

As regards PFS, only two RCTs (17, 18) comparing combination therapy with BCG alone in patients with high-risk NMIBC were available (*n* = 619). The meta-analysis demonstrated no significant difference in PFS between combination therapy and BCG alone (HR = 0.65, 95%CI: 0.25–1.68, *P* = 0.38), with high between-study heterogeneity (*P* = 0.02, *I*^2^ = 82%) (Figure 8). Subgroup analysis and publication bias detection were not performed due to limited number of enrolled studies for PFS.

### Overall Survival

**Figure 9:**
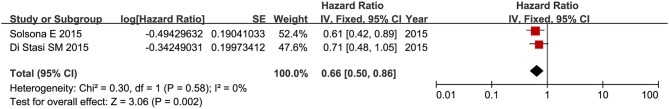
Forest plot of OS comparing combination therapy with BCG alone.

OS was compared between combination therapy and BCG alone in two trials (n = 619) (17, 18). The meta-analysis indicated that combination therapy was associated with a significantly better OS (HR = 0.66, 95%CI: 0.50–0.86, P = 0.002) than BCG alone without significant heterogeneity (P = 0.58, I^2^ = 0%) (Figure 9). As both two trials enrolled patients with high-risk NMIBC, same results were achieved in the subgroup of high-risk NMIBC patients with regard to OS. Considering that eligible studies were insufficient, we did not conduct other subgroup analyses or detect publication bias here.

### Disease-Specific Survival

**Figure 10:**
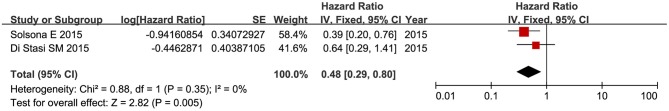
Forest plot of DSS comparing combination therapy with BCG.

When it came to DFS, two RCTs (17, 18) comparing combination therapy with BCG alone in patients with high-risk NMIBC were eligible (*n* = 619). Combination therapy appeared to confer longer DSS than BCG alone (HR = 0.48, 95%CI: 0. 29–0. 80, *P* = 0.005). No material heterogeneity was discovered (*P* = 0.35, *I*^2^ = 0%) (Figure 10). Same effect of combination therapy was discovered in the subgroup of high-risk NMIBC patients as that reported in the meta-analysis of DFS. Other subgroup analyses and the detection of publication bias were not performed due to limited studies.

### Side Effects

**Figure 11:**
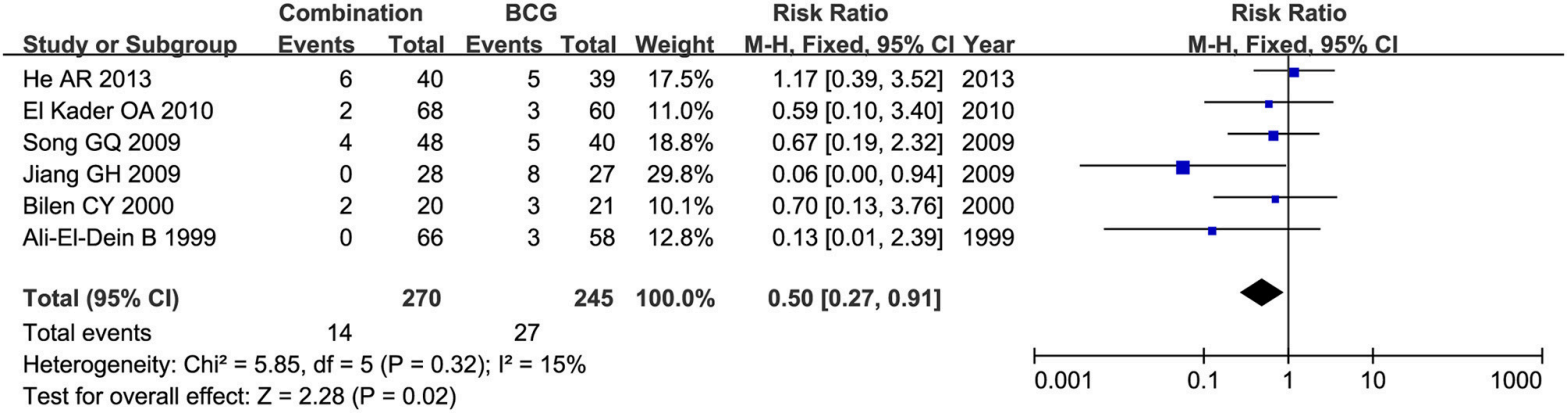
Forest plot comparing fever between combination therapy and BCG alone.

**Figure 12:**
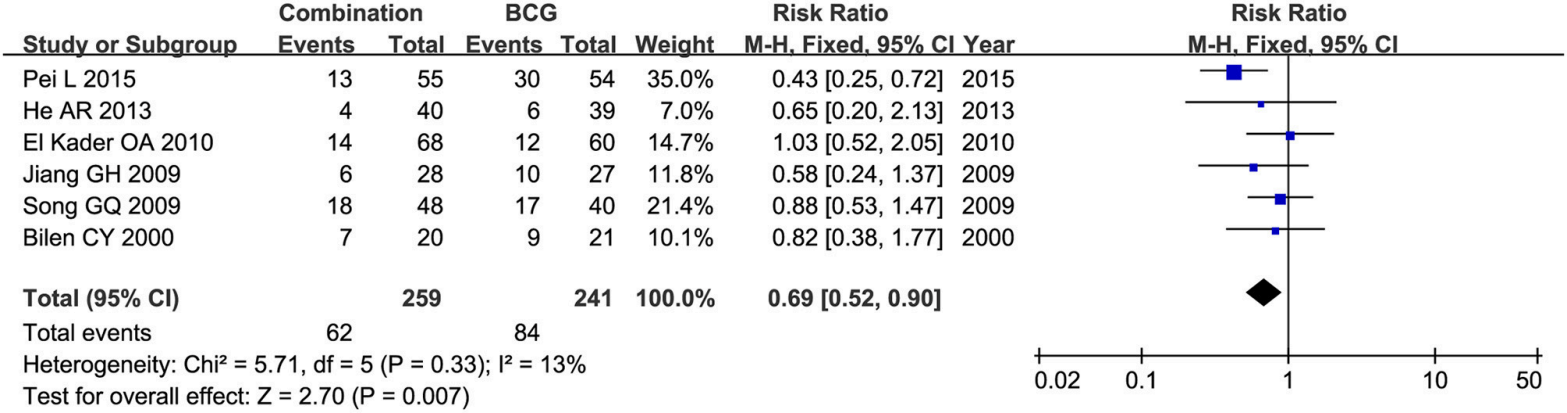
Forest plot comparing irritative bladder symptoms between combination therapy and BCG alone.

**Figure 13:**
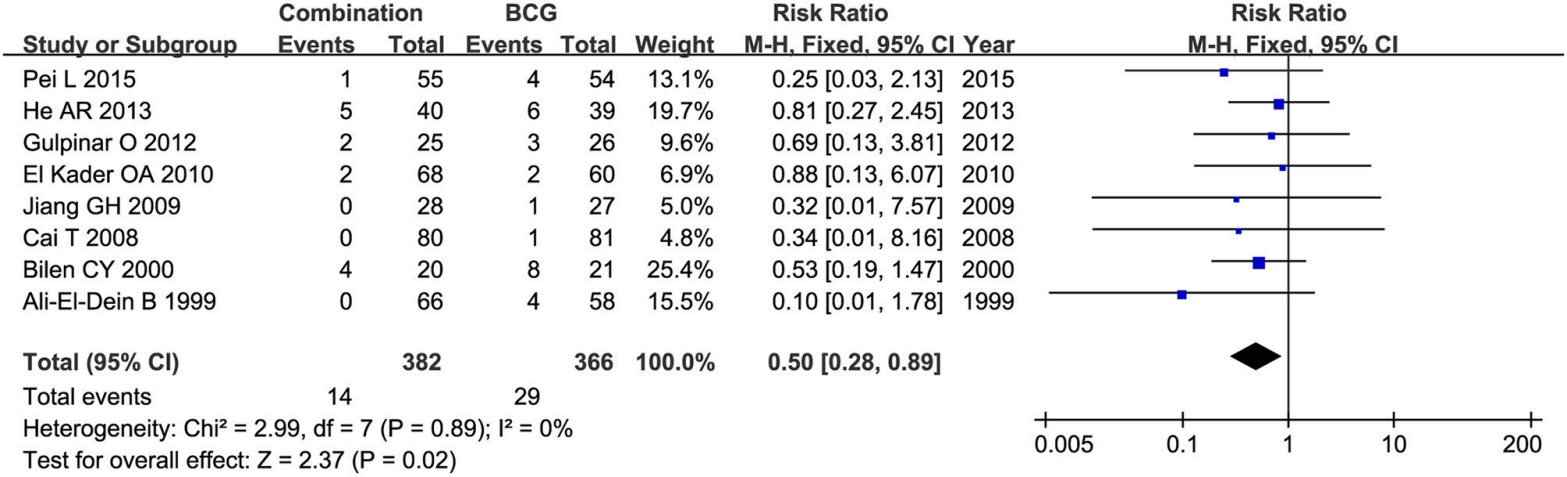
Forest plot comparing hematuria between combination therapy and BCG alone.

Toxicity comparison between combination therapy and BCG alone involved five studies (*n* = 814) (16, 18–20, 28). Combination therapy seemed not less toxic than BCG alone. The number of patients suffering fever was reported in 6 studies (20, 22–24, 27, 28), and pooled RR for combination therapy indicated significantly decreased rate of fever as compared with BCG alone (RR = 0.50, 95%CI: 0.27–0.91, *P* = 0.02) (Figure 11). Two studies (16, 20) mentioned gastrointestinal reaction and four (16, 26–28) stated cystitis, in which there were no significant differences between combination therapy and BCG alone (RR = 2.54, 95%CI: 0.61–10.60, *P* = 0.20; RR = 0.67, 95%CI: 0.29–1.54, *P* = 0.34). Irritative bladder symptoms was compared in 6 studies (16, 20, 22–24, 27) and hematuria in 8 (16, 20–23, 25, 27, 28). Combination therapy was related to lower rate of irritative bladder symptoms (RR = 0.69, 95%CI: 0.52–0.90, *P* = 0.007) (Figure 12) and hematuria (RR = 0.50, 95%CI: 0.28–0.89, *P* = 0.02) (Figure 13) than BCG alone.

**Figure 14:**
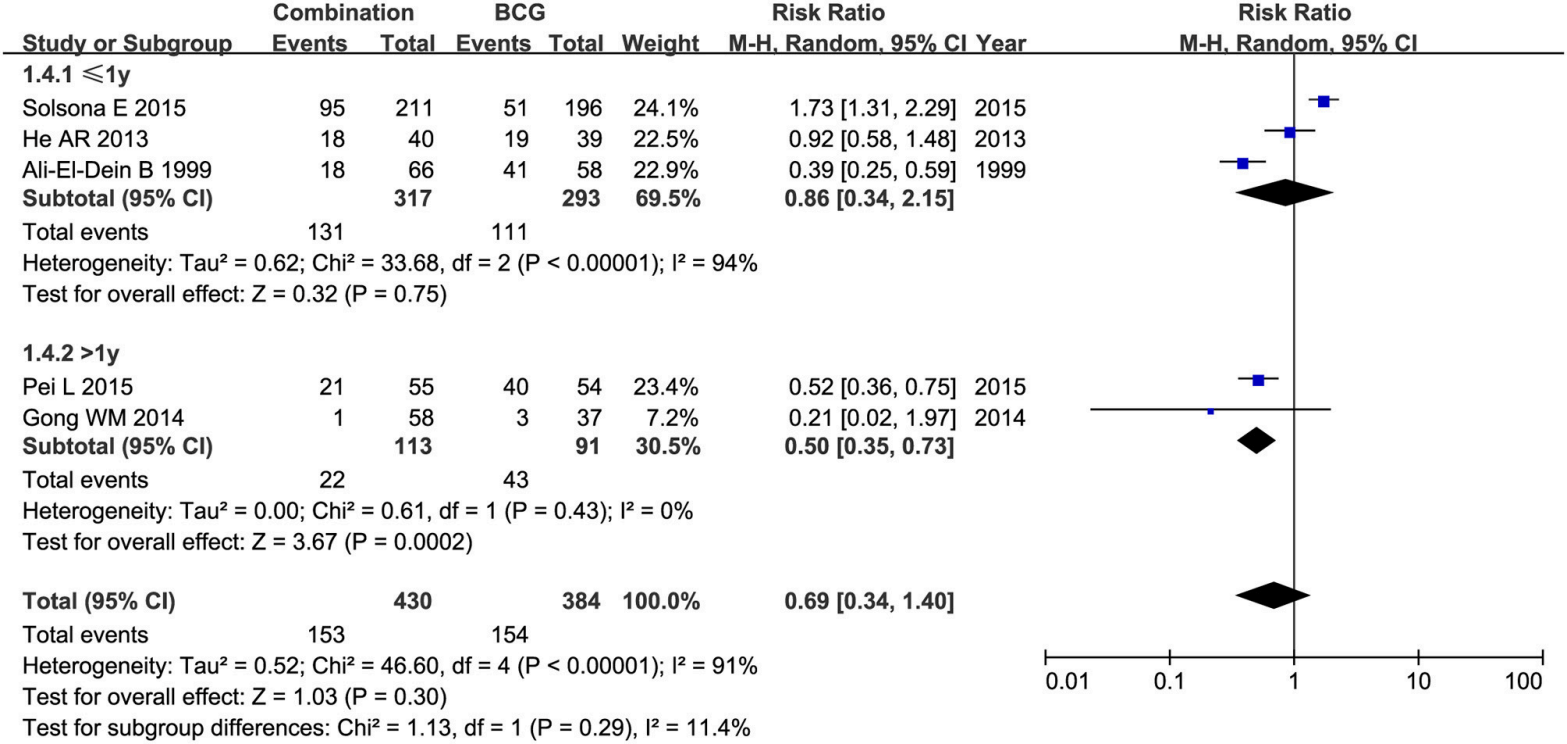
Forest plot comparing toxicity between combination therapy and BCG alone after subgroup analysis stratified by treatment duration.

Subgroup analyses of studies conducted in China demonstrated apparently decreased rate of irritative bladder symptoms for combination therapy than BCG alone (RR = 0.60, 95%CI: 0.43–0.83, *P* < 0.01). Studies conducted in foreign countries displayed a decline in hematuria rate for combination therapy compared with BCG alone (RR = 0.47, 95%CI: 0.23–0.98, *P* = 0.04). After stratification analysis by chemotherapeutic agent used in combination therapy, in EPI subgroup, combination therapy was more effective in preventing cystitis (RR = 0.47, 95%CI: 0.31–0.73, *P* < 0.01) and hematuria (RR = 0.36, 95%CI: 0.14–0.90, *P* = 0.03) than BCG alone; in THP subgroup, combination therapy decreased toxicity rate by 48% (RR = 0.52, 95%CI: 0.36–0.75, *P* < 0.01) compared with BCG alone. When treatment duration was more than 1 year, combination therapy appeared to reduce the rates of toxicity (RR = 0.50, 95%CI: 0.35–0.73, *P* < 0.01) (Figure 14), fever (RR = 0.06, 95%CI: 0.00–0.94, *P* = 0.05) and irritative bladder symptoms (RR = 0.46, 95%CI: 0.30–0.73, *P* < 0.01) compared with BCG alone. While focusing on patients with concurrent Tis, combination therapy was associated with lower rates of fever (RR = 0.08, 95%CI: 0.01–0.59, *P* = 0.01), cystitis (RR = 0.40, 95%CI: 0.26–0.63, *P* < 0.01), and hematuria (RR = 0.28, 95%CI: 0.09–0.88, *P* < 0.01) than BCG alone. In the subgroup of high-risk NMIBC patients, combination therapy revealed decreased rates in hematuria (RR = 0.36, 95%CI: 0.14–0.95, *P* = 0.04) and cystitis (RR = 0.47, 95%CI: 0.31–0.73, *P* < 0.01) as compared with BCG alone; however, no significant difference was discovered in toxicity (RR = 0.82, 95%CI: 0.19–3.59, *P* < 0.80) between combination therapy and BCG alone.

## Discussion

This meta-analysis was aimed to explore whether intravesical BCG in combination with chemotherapy is more effective and safer than BCG alone. We included 11 RCTs in the meta-analysis and found that the combination therapy was more advantageous than BCG alone in RFS, OS, and DSS among patients with NMIBC, with less side effects. No difference was observed in PFS between combination therapy and BCG alone. Subgroup analyses also indicated a significant reduction in the risk of tumor recurrence for combination therapy. Moreover, subgroup stratified by risk levels suggested that combination therapy was associated with better RFS, OS, and DSS in high-risk NMIBC patients, without increasing side effects.

BCG is most commonly used in intravesical immunotherapy for NMIBC and appears to be more effective than intravesical chemotherapy in preventing tumor recurrence and progression. Especially for those with high-risk NMIBC, BCG immunotherapy is considered as a gold-standard treatment (29). However, a large number of patients could not tolerate this agent because of its serious side effects (29). Thus, the compliance among these patients is poor in long-term follow-up, which will severely weaken the effect of BCG. Novel treatment is badly needed to improve the efficacy with less side effects. The combination of BCG with chemotherapeutic agent has ever been reported in some studies, with the expectation of improving anti-tumor effect and alleviating side effects through reducing BCG doses. Rintala et al. (30) once conducted a trial in patients with Tis to compare the efficacy of MMC plus BCG vs. MMC, and found that MMC plus BCG, without increasing side effects, was more effective than MMC. Subsequently, they reported a trial comparing the efficacy of alternating MMC plus BCG vs. MMC alone for recurrent papillary (stages Ta to T1) NMIBC, and similar efficacy was observed for the two treatment approaches without significant side effects (31). A randomized phase 3 trial comparing sequential intravesical therapy of MMC and BCG with MMC alone was performed by Witjes JA et al. (32) among patients with NMIBC; as a result, no differences in RFS and local side effects were detected between two methods, whereas systemic side effects were found to be more frequent in the MMC plus BCG group. A prospective RCT conducted by Cai et al. (25) in patients with high-risk NMIBC demonstrated no difference in RFS between the group receiving early single dose instillation of EPI followed by BCG and that with BCG alone. In a RCT reported by Solsona et al. (18), the combination of MMC and BCG reduced recurrence rate but showed more toxicity as compared with BCG alone. Conflicting effects of combination therapy in these researches could be explained by between-study heterogeneity in patients' characteristics, chemotherapeutic agents, BCG dose and follow-up duration. In addition, a large randomized phase 3 trial lasting for 7 years was designed to determine the efficacy and safety of combing BCG with MMC among patients with NMIBC (33, 34).

We also screened 4 meta-analyses published on this topic (11, 35–37). The meta-analysis by Houghton et al. (11) of 4 trials stated no advantage for the combination of intravesical chemotherapy with maintenance BCG overall, but suggested the possibility that the effect of combination therapy might depend on tumor stage with substantial benefit observed in patients with Ta or T1, but not in those with Tis, which indicated the need of exploring the efficacy of combination therapy on Ta or T1 bladder tumor. In our study, we only enrolled patients with Ta, T1 (with or without concurrent Tis), not those with Tis alone or recurrent bladder cancer. Other three meta-analyses on this topic were also screened. Deng et al. (35) reviewed 25 studies, and of the studies, 16 were RCTs, 4 retrospective comparative trials, 1 retrospective cohort study and 4 clinical series. Relevant data was extracted to calculate RRs and associated 95%CIs, and they found that the combination of BCG and MMC could significantly reduce the recurrence rate and cancer-specific mortality without more toxicities as compared with BCG; however, disease free survival and progression rate showed no significant differences between the two methods. In our study, only RCTs were included according to preset eligibility criteria, thereby only trials probably with high quality were incorporated. Meanwhile, we adopted HR to measure effect size in assessing prognosis, as opposed to RR used by Deng et al. Despite several differences in such aspects as patient characteristics, measurement of effect size and study design, the results of the two meta-analysis were partially similar. In our study, combination therapy could result in lower risk of recurrence with less side effects, but no difference in progression was observed, when compared with BCG alone. A meta-analysis of eight RCTs by Lan et al. (36) denoted significantly decreased recurrence rate for BCG in combination with MMC compared to BCG monotherapy, but no obvious differences were detected in progression rate, overall mortality or disease-specific mortality. This meta-analysis contained three same studies (17, 18, 21) as those in our study, other 5 studies were not included in our research for the reason that it did not meet our inclusion criteria. We included 6 studies conducted in China for our study. After stratified by original country (China vs. foreign countries), combination therapy appeared to be more effective both in China and foreign countries subgroups. Cui et al. (37) performed a systematic review and meta-analysis of 7 RCTs, and showed that patients with intermediate and high-risk NMIBC could benefit more from combination therapy than BCG alone in terms of recurrence and cystitis, with no difference in PFS. Subgroup analysis in this study also showed that patients with Ta or T1 (with or without concurrent Tis) could benefit more from combination therapy, but not those with Tis alone. Cui and colleagues enrolled 5 same trials (17, 18, 21, 25, 28) as those in our study. We recruited additional 8 RCTs comparing combination therapy with BCG monotherapy, totally 13 RCTs, and came to a similar conclusion that combination therapy was effective for patients with NMIBC but not for those with Tis alone. Despite substantial heterogeneity across the studies, similar results were still achieved, suggesting more efficacy and less side effects for combination therapy compared to BCG monotherapy.

Both the previous meta-analysis (37) and the present meta-analysis showed that combination therapy had no effect on reducing tumor progression as compared with BCG alone. A total of 5 studies were included in the meta-analysis performed by Cui et al. (37), and no significant effect was revealed on progression with substantial heterogeneity. Two RCTs (17, 18) were involved in our meta-analysis, one by Solsona et al. (18) reported no statistically significant difference between combination therapy and BCG monotherapy on PFS, the other by Di Stasi et al. (17) demonstrated a longer time to progression for patients receiving intravesical sequential BCG and electromotive MMC, which might suggest a possibility that electromotive delivery of chemotherapeutic agents could improve the efficacy of combination therapy. However, only 212 patients were included in this study and the potential effect of electromotive delivery should be interpreted with caution.

Both European Association of Urology (EAU) and American Urological Association (AUA) guidelines recommended that a second TURBT (also called re-TURBT) and a single immediate instillation of chemotherapeutic agent (SI) should be performed in specific cases after TURBT (7, 9). However, the evidences behind this recommendation were not consistent. We had also tried to explore whether re-TURBT, SI, and BCG induction or maintenance used in treatment would affect the results of this meta-analysis. However, the treatment schedule of each study varied a lot, such as BCG dose, chemotherapeutic agent used in combination therapy, treatment duration and the beginning time of adjuvant therapy after surgery. In addition, considering the limited number of eligible studies, we finally did not perform the meta-analysis and could not yet to confirm the effect of re-TURBT, SI, and BCG induction or maintenance on the results of this meta-analysis.

In this meta-analysis, subgroup analysis stratified by treatment duration indicated that combination therapy led to significantly lower rate of toxicity than BCG alone among patients treated for more than 1 year, which failed to be replicated in those treated for no more than 1 year. This may be explained by significant reduction in BCG dose, especially for patients undergoing combination therapy for a long time. BCG-related side effects usually occur in BCG maintenance treatment. It seemed that dose reduction was associated with decreases in side effects; although the effect of BCG dose on toxicity is unclear, it still suggests possible relationship of decrease in side effects with the reduction of BCG dose. In the outcomes of fever, cystitis, and hematuria, combination therapy was more advantageous than BCG alone among patients with concurrent Tis. Due to the small number of eligible studies, our findings still need to be further tested. We could not completely distinguish the risk levels for all patients included in this meta-analysis. However, most of them were diagnosed with intermediate- to high-risk NMIBC. Moreover, we extracted the studies involving patients with high-risk NMIBC and found that combination therapy was associated with better RFS, OS, and DSS in high-risk NMIBC patients. So we could speculate that combination therapy might be more effective among patients with intermediate- and high-risk NMIBC.

## Limitations

Although strict eligibility criteria were set to select publications, there were still several limitations. First of all, heterogeneity in treatment schedules, BCG doses or chemotherapeutic agents used in combination therapy, regimen choice of sequential or alternating treatments existed between the studies, In addition, included trials adopted different follow-up duration, ranging from 14.8 to 121 months. Finally, risk levels across patients also varied, thus we cannot evaluate the difference in efficacy between combination therapy and BCG alone for patients at specific risk levels. Those aforementioned problems may bias the conclusions of this meta-analysis. Despite such limitations, this meta-analysis was strictly performed via setting reasonable eligibility criteria and reviewing all available relevant data, thus comparing treatment effect between combination therapy and BCG alone.

## Conclusions

In summary, compared with BCG alone, combination therapy, without increasing side effects, was associated with lower recurrence as well as better OS and DSS among patients with NMIBC, but not among those with Tis alone or recurrent bladder tumor; some previous studies and this meta-analysis all showed that combination therapy had no effect on PFS. More high-quality RCTs are still required to confirm those conclusions.

## Author Contributions

Study was designed by X-HW and X-TZ. Searching of paper and data extraction was performed by DH and Y-HJ. Data was re-checked by HW. Statistical analyses was performed by DH and QH. Writing of the manuscript was performed by DH. X-HW and X-TZ reviewed the manuscript, and all authors read and approved the final manuscript.

### Conflict of Interest Statement

The authors declare that the research was conducted in the absence of any commercial or financial relationships that could be construed as a potential conflict of interest.
